# Out of Season and Out of Pattern: Winter Ehrlichiosis in a Heart Transplant Recipient With Possible Reactivation or Reinfection

**DOI:** 10.7759/cureus.109380

**Published:** 2026-05-21

**Authors:** Anum Fayyaz

**Affiliations:** 1 Medicine/Transplant Infectious Diseases, University of Louisville, Louisville, USA

**Keywords:** atypical presentation, diagnostic challenge, heart transplant, human monocytic ehrlichiosis (hme), immunosuppression

## Abstract

Human monocytic ehrlichiosis (HME) is a tick-borne infection caused by *Ehrlichia chaffeensis*, an obligate intracellular bacterium that infects monocytes and can lead to systemic inflammatory illness. Clinical manifestations typically include fever, malaise, cytopenias, transaminitis, and, in severe cases, neurologic or multiorgan involvement. Although HME is increasingly recognized in immunocompromised hosts, it remains uncommon in solid organ transplant recipients and is rarely reported outside peak seasonal exposure periods. In transplant patients, atypical presentations, baseline hematologic abnormalities, and the possibility of donor-derived or non-vector transmission can obscure the diagnosis and delay appropriate therapy. In addition, reactivation of ehrlichiosis following transplantation remains poorly characterized, with limited data available regarding its pathogenesis, diagnosis, and management. Here, we present a case of presumed reactivation of ehrlichiosis in a heart transplant recipient and discuss the diagnostic and therapeutic challenges associated with HME in immunosuppressed patients.

## Introduction

Human monocytic ehrlichiosis (HME) is a tick-borne infection caused by *Ehrlichia chaffeensis* [1], transmitted primarily by *Amblyomma americanum* [1]. We present a case of HME in a heart transplant recipient manifesting four weeks post-transplant during the winter months, well beyond the seasonal peak in tick activity and in the absence of new tick exposure, with clinical and serologic findings suggestive of reactivation of prior infection, a phenomenon not previously described in this population. This observation highlights a potentially underrecognized mechanism of disease and expands the clinical spectrum of ehrlichiosis in immunocompromised hosts. HME is otherwise well characterized in the general population, particularly in the south-central and southeastern United States during periods of peak tick activity. In addition to *Ehrlichia chaffeensis*, other species, including *Ehrlichia ewingii* and *Ehrlichia muris eauclairensis*, have been reported in humans, although data in transplant recipients remain limited [2,3].

Available literature suggests a propensity for more severe disease in immunocompromised hosts, with increased rates of complications such as acute respiratory failure, shock, and multiorgan dysfunction [3]. The clinical presentation is frequently nonspecific and may be further obscured by atypical features in this population, contributing to diagnostic delays. Although laboratory abnormalities--including leukopenia, thrombocytopenia, and transaminitis--are often described, they are not uniformly present [3].

In transplant recipients, diagnosis relies primarily on polymerase chain reaction (PCR) testing during the acute phase, as serologic responses may be delayed or attenuated due to immunosuppression [3]. Most reported cases are attributed to primary infection following recognized tick exposure, and data regarding alternative transmission pathways or reactivation remain sparse. Early empiric therapy with doxycycline is essential, as delays in diagnosis are associated with increased morbidity and mortality [2,3].

## Case presentation

A 66-year-old man underwent orthotopic heart transplantation in February 2025 after presenting with cardiogenic shock due to ischemic cardiomyopathy in the setting of multivessel coronary artery disease, requiring temporary mechanical circulatory support with an Impella device. He received induction therapy with methylprednisolone 1 g followed by tapering steroids and was maintained on standard institution-specific immunosuppression for heart transplant recipients, including mycophenolate 1 g BID and tacrolimus (target trough 10-12 ng/mL). No rejection episodes or augmentation of immunosuppression occurred. His pre-transplant evaluation and perioperative course were unremarkable for infectious concerns, and he was discharged home approximately one week post-transplant with routine follow-up.

Approximately four weeks after transplantation, he presented with a three-day history of fever and dry cough. He reported temperatures up to 102°F without chills or night sweats and denied sputum production or other respiratory, gastrointestinal, or systemic symptoms. His early post-transplant course had been uncomplicated, and he reported strict adherence to infection prevention measures, remaining predominantly indoors with no outdoor activity or animal exposures.

On presentation, he required new supplemental oxygen at 2 L/min via nasal cannula, whereas he had previously been stable on room air. Psoas myositis was attributed to intramuscular hemorrhage in the setting of thrombocytopenia, with negative microbiologic evaluation including pneumonia workup. Physical examination was otherwise unremarkable. Laboratory evaluation revealed leukopenia with neutrophil predominance and profound lymphopenia (Table 1). He was also found to have hyponatremia, with otherwise preserved renal and hepatic function (Table 1).

**Table 1:** Laboratory trends from presentation to 48 hours

Parameter	Presentation	48 hours	Reference Range	Trend (Change)
WBC (×10⁹/L)	2.2	1.6	4.0–11.0	↓
Neutrophils (×10⁹/L)	1.9	0.9	1.5–7.5	↓
Lymphocytes (×10⁹/L)	0.2	0.2	1.0–3.0	→
Hemoglobin (g/dL)	10	8.5	13.5–17.5	↓
Platelets (×10⁹/L)	151	75	150–400	↓
Sodium (mmol/L)	126–128	121	135–145	↓
Potassium (mmol/L)	4.7	4.7	3.5–5.0	→
Creatinine (mg/dL)	1.14	1	0.7–1.3	→
eGFR (mL/min/1.73m²)	71	80	>60	→
Total bilirubin (mg/dL)	—	0.5	0.1–1.2	→
Alkaline phosphatase (U/L)	93	112	40–130	→
AST (U/L)	41	155	10–40	↑
ALT (U/L)	40	82	7–56	↑

Chest CT demonstrated a new right lower lobe consolidation (Figure 1). He was started on antimicrobials in consideration of legionella pneumonia. Infectious workup for atypical pneumonia was obtained and was negative. The cardiac function was normal.

**Figure 1:**
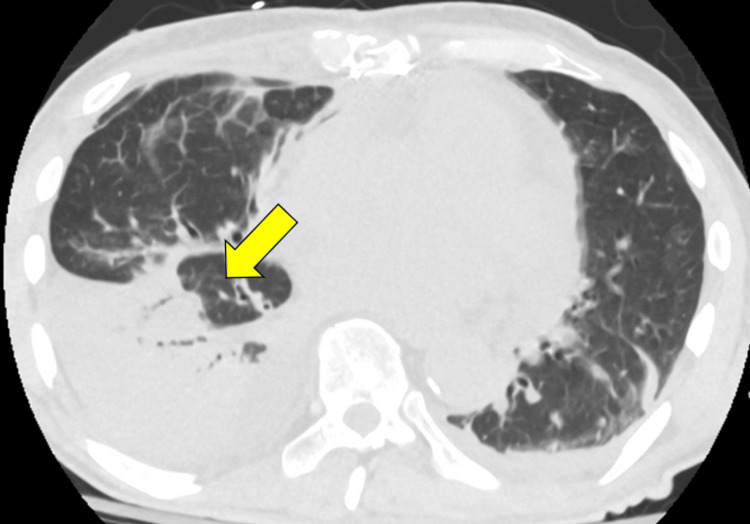
Consolidation in the basilar right lower lobe likely suspicious for superimposed pneumonia

Over the ensuing 36-48 hours, he clinically deteriorated with new acute confusion and progressive cytopenias, including worsening leukopenia, anemia, and thrombocytopenia (Table 1). His hyponatremia also worsened, and repeat labs showed rising transaminase levels (Table 1). In the setting of evolving multisystem involvement, CT imaging of the abdomen and pelvis was obtained, revealing left psoas muscle inflammation concerning for myositis (Figure 2).

**Figure 2:**
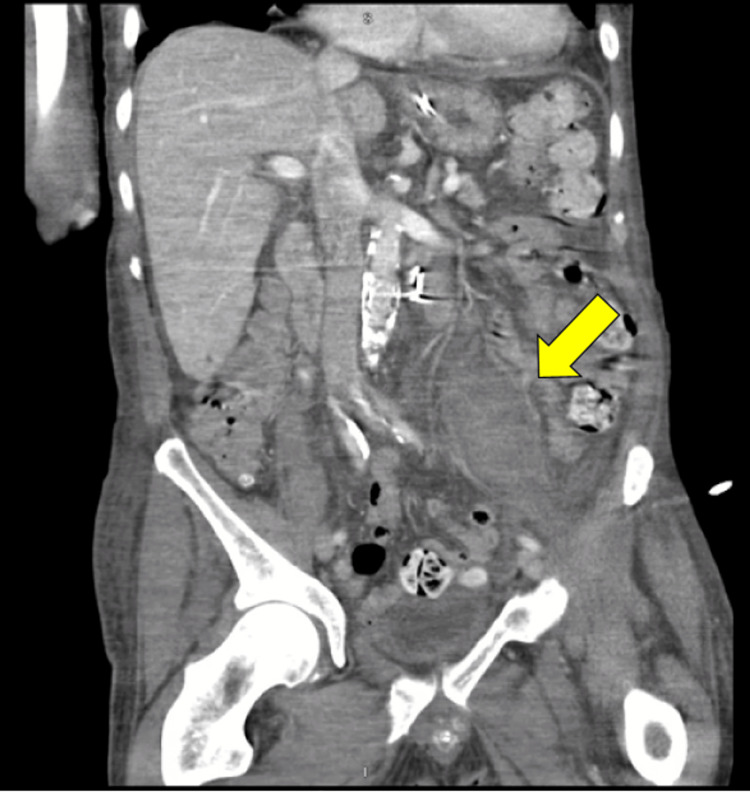
CT imaging of the abdomen and pelvis revealing left psoas muscle inflammation concerning for myositis

At that stage, the differential diagnosis was expanded beyond *Legionella pneumonia* to include tick-borne infection, and empiric doxycycline therapy was initiated. Interventional radiology evaluation of the psoas lesion subsequently demonstrated a blood collection (hematoma) rather than an abscess, consistent with his evolving hematologic abnormalities and clinical course.

Given ongoing diagnostic uncertainty, plasma microbial cell-free DNA testing was performed and returned positive for *Ehrlichia chaffeensis* within 48 hours, confirming human monocytic ehrlichiosis as the etiology of his presentation. He demonstrated clinical improvement over the subsequent 48 hours after initiation of doxycycline.

Given the temporal proximity to transplantation, concern was raised for a possible donor-derived infection, and the case was reported in accordance with Organ Procurement and Transplantation Network (OPTN) policy (Policy 15.4.A and Policy 15.5.B) to facilitate evaluation of other recipients; all other organ recipients from the same donor remained clinically well, and the donor tested negative for HME [4,5]. The infection was also reported to the Kentucky Department of Public Health; however, a definitive source could not be established, and the possibility of transfusion-associated acquisition could not be confirmed. Samples from the patient’s pre- and post-transplant course were submitted to the CDC for further investigation.

The CDC’s retrospective review demonstrated that the recipient had a negative *Ehrlichia* PCR result but positive *Ehrlichia *IgG before transplantation (Table 2), with a subsequent fourfold rise in serology and conversion to PCR positivity at the time of illness four weeks post-transplant (Table 3). Further history revealed remote exposure to ticks during the summer of 2024, approximately one year before transplantation. The patient did not recall any associated illness or seek medical evaluation at that time. He had since relocated and reported no subsequent tick exposures. He had not exhibited symptoms during the pre-transplant evaluation. Most likely, he had reactivation of HME post-heart transplant.

**Table 2:** CDC tests of recipient blood before heart transplantation

Test	Result
EHR1 *Ehrlichia spp.* real-time PCR	Not detected
ECH2 *Ehrlichia chaffeensis* real-time PCR	Not detected
IgG – *Ehrlichia chaffeensis* (titer)	128

**Table 3:** CDC tests of recipient blood four weeks after heart transplantation

Test	Result
EHR1 *Ehrlichia spp.* real-time PCR	Detected
ECH2 *Ehrlichia chaffeensis *real-time PCR	Detected
IgG – *Ehrlichia chaffeensis *(titer)	512

## Discussion

When evaluating infections in immunocompromised hosts, particularly solid organ transplant recipients, several key factors must be considered. A post-transplant timeline is often used to approximate immune status and infectious risk (Figure 3). In the early post-transplant period (within the first month), patients are susceptible to donor-derived infections. Between one and six months post-transplant, the risk of infection is largely driven by the intensity of immunosuppression and the resulting impaired host defenses. Heart transplant recipients, by the nature of their required immunosuppressive regimens, remain highly immunocompromised, and rejection risk often necessitates sustained immune suppression [5].

**Figure 3:**
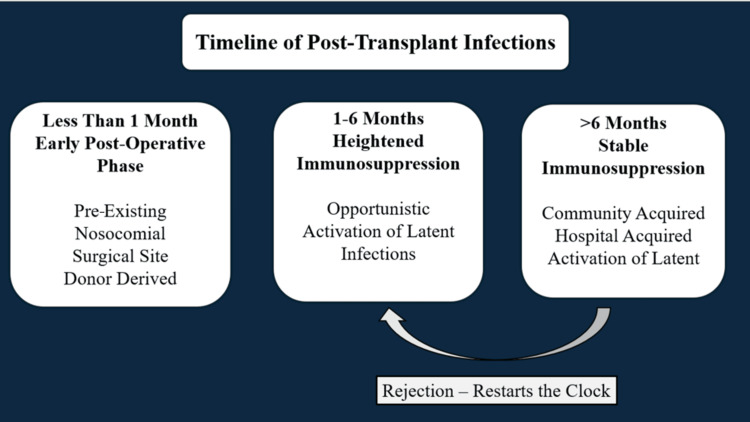
Timeline of post-transplant infections

Data regarding reactivation of HME in solid organ transplant recipients remain extremely limited, and the distinction between reactivation and newly acquired infection is often difficult to establish. Nonetheless, our case raises the possibility of HME reactivation in the setting of intense immunosuppression, highlighting an area that remains poorly characterized and warrants further investigation. To date, approximately 167 cases of HME have been reported in solid organ transplant recipients, the vast majority occurring in kidney transplant patients [3]. Outcomes in this population have been poor in a subset of cases, often due to either donor-derived transmission or delayed diagnosis. A high index of suspicion is essential for timely recognition, as donor exposure history is frequently incomplete and may not reliably identify risk. Additionally, there are currently no data supporting the use of primary prophylaxis for HME in patients with prior exposure. Hence, timely diagnosis is imperative.

In a retrospective study evaluating the impact of immunosuppression on HME, 15 transplant recipients were compared with non-immunocompromised hosts [2]. The authors noted that transplant patients most commonly presented with fever and neurologic symptoms, often raising concern for meningitis or meningoencephalitis. The most frequent laboratory abnormalities included cytopenias and elevated creatinine, findings that are also commonly observed in transplant recipients at baseline due to chronic immunosuppression and comorbidities. As a result, post-transplant patients often have pre-existing hematologic abnormalities, which can obscure the recognition of acute infectious processes and contribute to delays in diagnosis. Hence, clinical syndromes need high diagnostic vigilance [2].

Another important consideration is that, although diagnostic capabilities for tick-borne infections have advanced from serology alone to include PCR-based and multiplex PCR assays, many of these tests are still performed at reference laboratories, which can contribute to diagnostic delays. Therefore, in the appropriate clinical context and under infectious disease guidance, cell-free DNA testing has an important role in the diagnosis of zoonotic and donor-derived infections, particularly when rapid etiologic confirmation is needed [3,6].

There is also limited guidance regarding the optimal duration of treatment, as clinical presentations, timing relative to transplantation, and degree of immunosuppression vary across different organ transplant populations. Most reported cases have been successfully treated with approximately 14 days of therapy; however, given the clinical complexity of these patients and the lack of definitive prospective studies, it is difficult to generalize this approach to all cases. Additionally, this case raises the question of secondary prophylaxis following infection, which remains an area that has not been well defined or systematically studied in solid organ transplant recipients [3].

## Conclusions

In conclusion, the evaluation and management of tick-borne infections in solid organ transplant recipients require a high degree of clinical suspicion and an appreciation of the time-dependent nature of post-transplant infectious risk. Diagnostic interpretation is often complicated by baseline abnormalities related to chronic immunosuppression, particularly cytopenias and renal dysfunction, which can obscure early recognition of infection. Although diagnostic modalities have advanced from serology to PCR and multiplex PCR testing, many of these assays remain reference-laboratory based, potentially delaying definitive diagnosis. In this context, emerging tools such as cell-free DNA testing may have a valuable role in rapidly identifying zoonotic and donor-derived infections when used judiciously under infectious disease guidance. Importantly, this case also highlights a previously unreported consideration of potential reactivation of tick-borne infection in the post-transplant setting, a phenomenon that has not been well described in the existing literature and warrants further investigation.
